# A bibliometric analysis of global publications on graft-versus-host disease research

**DOI:** 10.1097/MD.0000000000029634

**Published:** 2022-07-08

**Authors:** Xuemiao Huang, Taiwei Wang, Wanting Zu, Tianxin Xu, Lin Du, Yiming Wang, Wenbo Nie, Lisheng Wang

**Affiliations:** a School of Nursing, Jilin University, Changchun, China.

**Keywords:** bibliometric analysis, citation network, collaborative network, graft-versus-host disease, thematic trends

## Abstract

**Background:**

Graft-versus-host disease (GVHD) is a fatal complication of hematopoietic stem cell transplantation and is an enormous burden on the patient economy and related health systems. Nevertheless, only a few bibliometric studies have examined the direction of research and the major findings within the field.

**Methods:**

Statistical and visualization bibliometric analysis was performed in April 2021. Our research data were retrieved from the Web of Science using an advanced search strategy. We then used bibliometric analysis to determine the current general research direction and trend of publications and established the most prolific and distinguished authors, institutions, countries, funding agencies, and keywords in GVHD research. We employed VOSviewer (Leiden University, Leiden, Netherlands), Microsoft Excel (Microsoft, Redmond, State of Washington), and GunnMap (https://lert.co.nz/map/) to retrieve, integrate, and visualize the results.

**Results:**

Overall, 15,378 publications from 500 journals were extracted from the Institute for Scientific Information (ISI) Web of Science Core Collection database based on our analysis, of which the United States and the Fred Hutchinson Cancer Research Center were the most prolific countries and institutions, respectively. Moreover, we identified future research trends and the current status of GVHD research based on the top 10 most cited articles. Finally, influential authors’ analysis demonstrated that Blazar, BR were both the most productive and most cited among all authors.

**Conclusion:**

Our study provides an exhaustive and objective overview of the current status of GVHD research. This information would be highly beneficial to anyone seeking information on GVHD and would serve as a reference guide for researchers aiming to conduct further GVHD research.

## 1. Introduction

Graft-versus-host disease (GVHD) is a severe immune response^[1]^ to allogeneic hematopoietic stem cell transplantation (HSCT).^[2,3]^ It is not commonly observed after whole organ transplantation, autologous HSCT, or blood transfusion.^[4–6]^ The incidence of donor lymphocytes interacting with foreign antigens to promote inflammation can be as high as 40%–60% in patients undergoing HSCT.^[7]^ Moreover, this potentially fatal disease carries a mortality rate as high as 15%.^[8,9]^ Therefore, it is essential to provide acute HSCT recipients with prophylaxis to prevent GVHD.^[10]^ Chronic GVHD is a common complication among long-term survivors of allogeneic HSCT.^[11]^ With a steep increase in long-term survivors, the incidence of delayed complications, emerging years after HSCT, is becoming a common concern.

Given the overall burden of GVHD, recent decades have witnessed an explosion in both clinical and basic research in this field. As a result, certain journals and institutions have emerged as dominant contributors to GVHD research. In the Web of Science (WoS) database, the search term “graft-versus-host disease” returned over 20,000 articles published since 1977. With great advancements in this field, the area of GVHD research has become increasingly diversified.

“Bibliometrics” is a critical tool for the analysis of the status quo in research fields.^[12]^ This concept was initially introduced by Pritchard (1996) as “the application of mathematical and statistical methods to books and other media.” Using this tool, one can assess particular parameters, such as the author, country of publication, and research design. Over the last few decades, bibliometric analysis has received much recognition within the scientific community, especially with regard to its ability to present current trends in a given research within a specified time.^[13]^ Nevertheless, very few studies have employed bibliometric analysis to extract such information. The goal of this work was to analyze the scientific output in the field of GVHD and track its evolution worldwide based on the information gathered from the WoS database. A systemic investigation of articles involving GVHD, using statistical and visualization bibliometric analysis (SAVBA), can provide crucial and detailed information to scientists involved in GVHD research.

## 2. The study

### 2.1. Aims

Our goal was to assess the level and quality of GVHD research performed by authors, journals, funding agencies, and institutions worldwide using SAVBA. Moreover, we used keyword co-word analysis to determine the overall research direction and interest. Furthermore, we performed a deep evaluation of the keywords and research design of prolific giants in the field of GVHD research and presented a summary of their work to better elucidate the direction, type, and areas of study, as well as historical and emerging evidence, and more regarding GVHD research. Our work will be particularly beneficial as an overview for scientists in the field of GVHD research.

### 2.2. Design

Published articles on GVHD research were eligible for SAVBA. The bibliometric information of the articles (nationality, affiliations, authors, year of publication, publishing journal, title, abstract, keywords, and document type) was analyzed using descriptive techniques and bibliometric mapping.

### 2.3. Participants

We collected information from the WoS database for the analysis. Hence, no subjects were selected for this study.

### 2.4. Data collection

We collected all data from the ISI WoS Core Collection database (ISI-WoS-CCD) and the InCites Journal Citation Reports (JCR) on April 1, 2021. To certify data accuracy, we employed synonyms and related terminology during our keyword search. Examples of keywords used for data collection are as follows: ((“graft versus host disease”) OR (“graft vs. host disease”) OR (“graft-versus-host disease”) OR (“graft-versus-host-disease”) OR (“GVHD”) OR (“graft vs host disease”)) AND Language =English AND Document type = Articles.” We also adjusted the search parameters such that we received all GVHD-related articles from the establishment of the database until April 2021.

The inclusion criteria of this study are as follows: the database to be included is the CCD of WoS, Publication date: April 1, 2021, and refine the Article type into Article. The retrieved literatures were excluded according to the following criteria: conference abstracts, action research, book reviews, news, materials, nonpublished literatures, or literatures requiring correction and in duplicate publications, only newly published studies were included.

### 2.5. Ethical considerations

Owing to the noninvolvement of human participants, this study did not require ethical approval.

### 2.6. Data analysis

Java program VOSviewer and Microsoft Excel software were employed for analysis and graphic processing of the nationalities, affiliations, funding agencies, publication years, journals, authors, keywords, and most-cited articles. Simultaneously, VOSviewer was utilized for the extraction and generation of bibliometric illustrations to visualize, compute, and analyze the co-occurrence network of terminologies collected from the title and abstract of the publications, the collaboration axis between countries, and the cocitation and bibliographic coupling of network relations among authors.^[14]^ In addition, GunnMap (http://lert.co.nz/map/) was utilized to produce a world map depicting publication distribution.

### 2.7. Validity and reliability/rigor

All citation information was exported from the ISI WoS database in TXT format and imported into VOSviewer and Microsoft Excel. Articles were included and excluded independently by 2 researchers. However, when faced with discrepancies, a third researcher was employed to reconcile the differences. All analyses were performed using quantitative data, thereby increasing the reliability of the conclusions.

## 3. Results

### 3.1. Descriptive analysis

**Figure 1.**
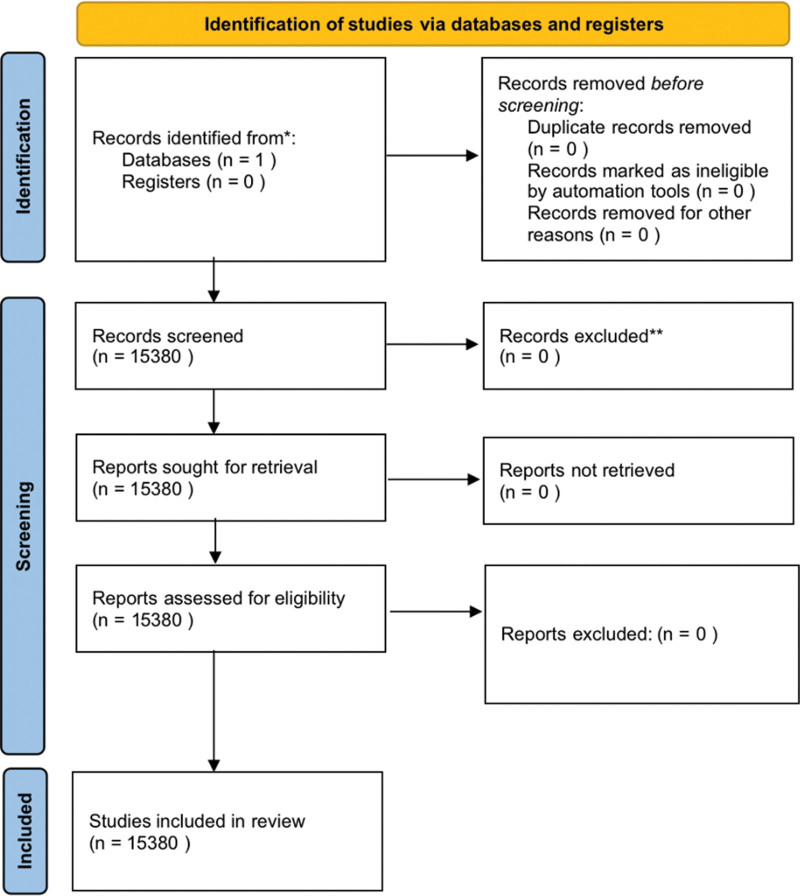
The process of literature search has been indicated in the flow diagram.

From the inception of the database until April 2021, 24,241 English publications were available in the ISI-WoS-CCD, including all different document types. Among them, 15,380 English articles were selected for this study based on the inclusion and exclusion criteria, which represented 63.44% of the total literature publications, indicating that these articles were part of the main document type (Fig. 1). The remaining document types fell under the categories of the abstracts, reviews, conference articles and letters categories, as well as republished articles, and are excluded from our analysis.

#### 3.1.1. Publication distribution across time and countries.

**Figure 2.**
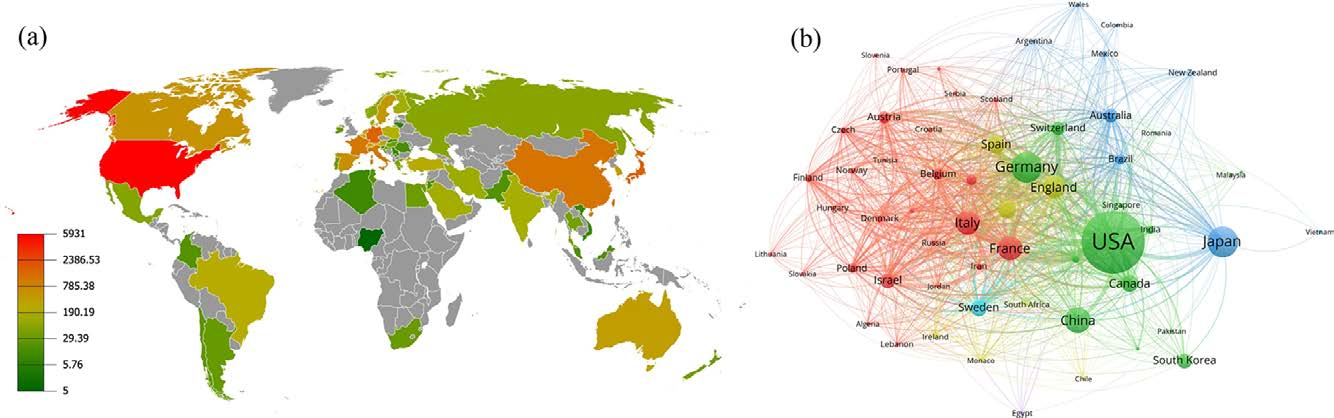
(A) Global geographic distribution of the total number of articles by country. (B) Network visualization map of country coauthorship; the countries were classified into different clusters with various colors automatically.

Eligible publications were published in 86 countries across all continents (Fig. 2A). Among them, 5 countries published 5 to 9 articles over the years, whereas 28 countries published no more than 100 articles. The color scale was adjusted to display the publication frequency, with red representing abundant publications and green representing fewer publications. Next, we applied country coauthorship network visualization analysis to determine the level of international collaboration (Fig. 2B). United States ranked number 1 in intercountry collaborations. In the bibliometric diagram, the countries are separated into 6 clusters. The size of each circle corresponds to the contributions of the cluster in GVHD research, whereas the thickness of the lines connecting different countries corresponds to the level of collaboration between the 2 nations.

**Figure 3.**
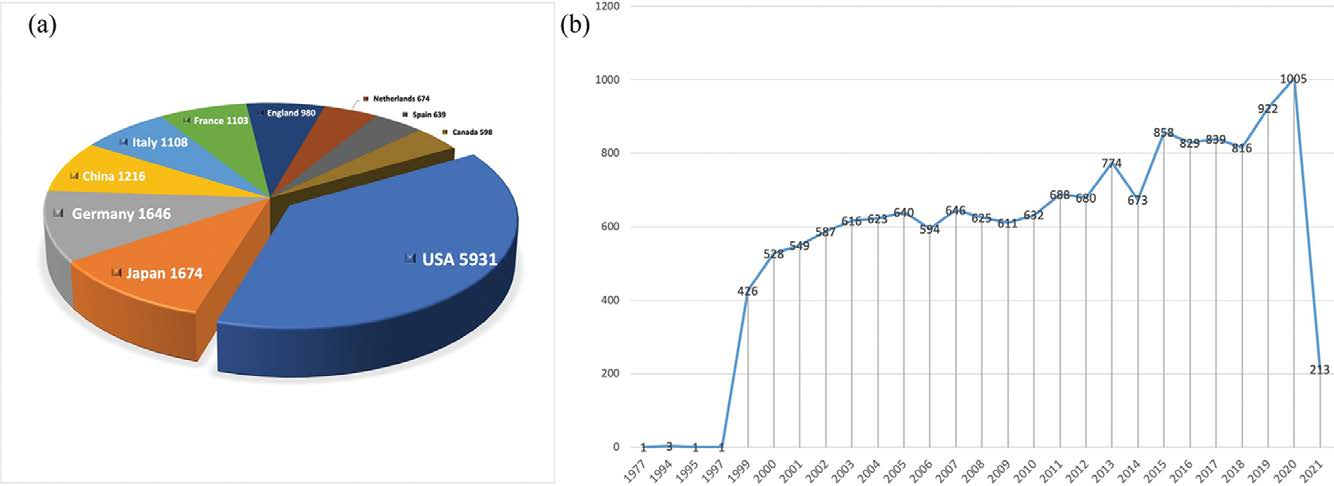
(A) The top 10 prolific countries based on the number on publications related to GVHD. (B) The growth of total number about the articles on GVHD research. GVHD = graft-versus-host disease.

Figure 3A lists the top 10 countries with the largest number of publications in the field of GVHD research. The most productive country by April 2021 was the United States (n = 5931, 38.56%). This is likely due to its ranking as a global scientific leader owing to its massive research efforts.^[15,16]^ The United States contains numerous high-level national research institutions that produce high-quality articles with excellent reputations. Reputation plays a significant role in research, and therefore, most researchers outside the United States aim to collaborate with research labs/institutions within the United States.^[17]^ The next most productive countries were Japan (n = 1674, 10.88%) and Germany (n = 1646, 10.70%). Slightly, less prolific were countries like China (n = 1216, 7.91%) and Italy (n = 1108, 7.20%), followed by France (n = 1103, 7.17%), England (n = 980, 6.37%), the Netherlands (n = 674, 4.38%), Spain (n = 639, 4.15%), and Canada (n = 598, 3.89%). The growth trend of the articles involved in GVHD research is illustrated in Figure 3B, which clearly shows that very few articles were published before 1999, and then, the trend of publication increased remarkably year by year.

#### 3.1.2. Publication analysis based on journals.

In total, 338 journals containing publications on GVHD were included in this study. Using 6 main indexes, including Journal Impact Factor (JIF), total article, Total Global Citation Score (TGCS), Indexed Categories, Journal Country, and JIF Quartile, we generated a top 10 journal list, based on the journals that published the most GVHD research (Table 1). The WoS categories represent journal disciplines. JIF is an influential index that evaluates the academic caliber of journals and was retrieved from JCR in 2020. The JIF quartile refers to the JIF quartile involving different journals and evaluates the publication distribution of a specific entity, namely, a country, institution, research group, or individual, among journals of different fields.^[17–19]^ As depicted in Table 1, the most productive journal based on our analysis was Biology of Blood and Marrow. The number of total articles was 2009 (13.073%) until April 2021. The top 10 journals focused on immunology, hematology, transplantation, oncology, and surgery, depending on the corresponding InCites JCR. Although some of the top 10 journals published fewer articles, they played a significant role in terms of TGCS. For example, both the British Journal of Hematology (12,326) and the Journal of Immunology (12,990) had higher TGCS but relatively fewer publications. Moreover, more than 50% of the top 10 journals exhibited an official JIF of >3.0, the highest being Blood, with a JIF of 17.794. Meanwhile, 5 of the top publishers were from the United States and 2 from England. The remaining were from Japan, Germany, and Italy. In terms of quartile in category, half of these publishers were in Q1.

**Table 1:** Quantitative measurement of journals publishing research on graft versus host disease.

Rank	Journals	JIF(R)	TA (%)	TGCS	Indexed categories	JC	JIF quartile
1	BIOLOGY OF BLOOD AND MARROW TRANSPLANTATION	3.853	2009 (13.073)	54499	Immunology; Haematalogy; Transplantation	United States	Q2
2	BONE MARROW TRANSPLANTATION	4.725	1293 (8.414)	32528	Immunology; Oncology; Haematalogy; Transplantation	England	Q1
3	BLOOD	17.794	1195 (7.776)	115331	Haematalogy	United States	Q1
4	TRANSPLANTATION	4.546	454 (2.954)	14158	Immunology; Transplantation; Surgery	United States	Q1
5	BRITISH JOURNAL OF HAEMATOLOGY	5.518	352 (2.291)	12362	Haematalogy	England	Q1
6	INTERNATIONAL JOURNAL OF HEMATOLOGY	2.245	324 (2.108)	3125	Haematalogy	Japan	Q3
7	JOURNAL OF IMMUNOLOGY	4.886	315 (2.05)	12990	Immunology	United States	Q2
8	ANNALS OF HEMATOLOGY	2.904	222 (1.445)	1870	Haematalogy	Germany	Q2
9	HAEMATOLOGICA	7.116	204 (1.328)	6097	Haematalogy	Italy	Q1
10	TRANSPLANTATION PROCEEDINGS	0.784	185 (1.204)	1696	Immunology; Transplantation; Surgery	United States	Q4

JC = journal country, JIF = journal impact factor, JIF = journal impact factor, TA = total articles, TGCS = Total Global Citations Score.

#### 3.1.3. Publication analysis according to institutions and funding agencies.

We further examined the contribution of the institutions to GVHD research. A total of 1000 institutions published articles on GVHD. The top 10 institutions involved in GVHD research are summarized in Table 2. Most of the top 10 institutions were from the United States, confirming the presence of significant professional research organizations within this country. Meanwhile, there is only 1 institution in the Netherlands. Moreover, among the institutions that produced the most articles in GVHD research, the Fred Hutchinson Cancer Research Center ranked number 1. As of April 1, 2021, this institution had 755 publications with the largest citation (46507) among all articles. The University of Minnesota and University of Washington had 627 and 607 publications, respectively. Interestingly, although Stanford University did not have the most publications, its citations were remarkably high compared to other institutions, even the Dana-Farber Cancer Institute.

**Table 2:** Quantitative measurements of organizations publishing research on graft versus host disease.

Rank	Organization	N (%)	Citation	Country
1	Fred Hutchinson Cancer Research Center	755 (4.91)	46507	United States
2	University of Minnesota	627 (4.08)	43862	United States
3	University of Washington	607 (3.95)	36635	United States
4	Medical College of Wisconsin	417 (2.71)	26518	United States
5	Dana-Farber Cancer Institution	321 (2.09)	19592	United States
6	Stanford University	320 (2.08)	24385	United States
7	Harvard University	298 (1.94)	18200	United States
8	Memorial Sloan-Kettering Cancer Center	287 (1.87)	15556	United States
9	University of Michigan	275 (1.79)	17586	United States
10	Leiden University	256 (1.66)	14198	The Netherlands

The top 10 funding bodies are listed in Table 3, with 5 located in the United States. The United States Department of Health Human Services and the National Institutes of Health (NIH), United States, endorsed 3600 and 3597 studies, respectively, constituting 46% of all studies. Nearly 80% of the studies were obtained from these 5 funding bodies. Funding agencies from Japan, China, and Europe also supported many studies. Together, these funding agencies offer crucial assistance for the advancement of GVHD research.

**Table 3:** Quantitative measurements of funding agencies publishing research on graft versus host disease.

Rank	Funding agency	Location	N (%)
1	UNITED STATES DEPARTMENT OF HEALTH HUMAN SERVICES	United States	3600 (23.422)
2	NATIONAL INSTITUTES OF HEALTH NIH USA	United States	3597 (23.403)
3	NIH NATIONAL CANCER INSTITUTE NCI	United States	2501 (16.272)
4	NIH NATIONAL HEART LUNG BLOOD INSTITUTE NHLBI	United States	1472 (9.577)
5	NIH NATIONAL INSTITUTE OF ALLERGY INFECTIOUS DISEASES NIAID	United States	1081 (7.033)
6	MINISTRY OF EDUCATION CULTURE SPORTS SCIENCE AND TECHNOLOGY JAPAN MEXT	Japan	599 (3.897)
7	NATIONAL NATURAL SCIENCE FOUNDATION OF CHINA NSFC	China	558 (3.63)
8	JAPAN SOCIETY FOR THE PROMOTION OF SCIENCE	Japan	548 (3.565)
9	GRANTS IN AID FOR SCIENTIFIC RESEARCH KAKENHI	Japan	501 (3.26)
10	EUROPEAN COMMISSION	Europe	442 (2.876)

### 3.2. Research focused analysis by co-occurrence of keywords and research category

**Figure 4.**
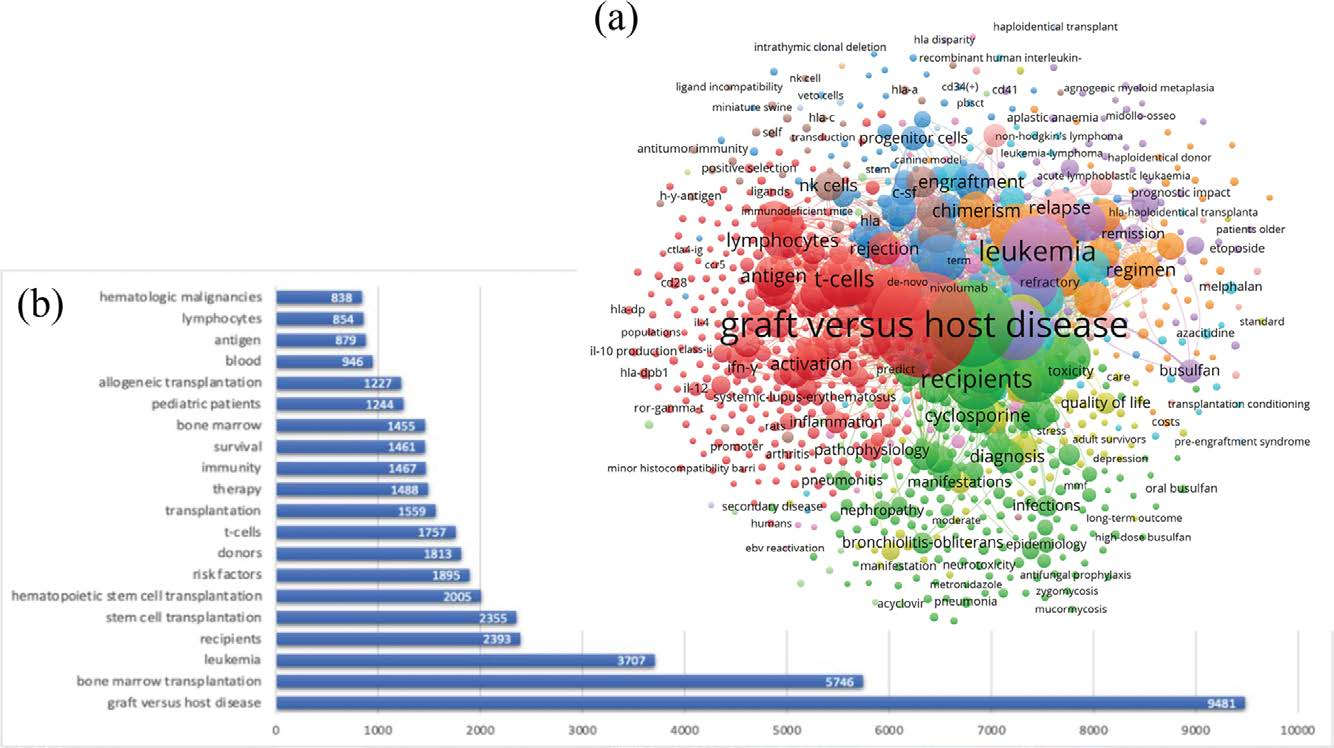
(A) Co-occurrence map of most frequently encounter from the titles and abstracts of retrieved articles. (B) The top 20 most frequency keywords.

Keywords play a major role in the analysis of data sources and reveal the main contents of existing research, while summarizing information regarding terms, goals, and methods, and themes of articles.^[20–22]^ Keyword co-occurrence refers to >2 keywords appearing in the same article simultaneously.^[23]^ This analysis can establish hot topics and track research frontier transitions in the scientific domain.^[24,25]^ Keywords were expected to be preprocessed because there could be variations in the same word, which could affect the final analysis. Therefore, to prevent bias in the expressions of publications, we employed keywords, such as, graft versus host disease,” “graft-versus-host disease,” and “GVHD” as variations of the same word. To perform our analysis, we extracted keywords from the titles and abstracts of eligible articles, and keywords with an occurrence of >10 were entered into the final analysis. All terminologies were selected by the researchers, and a network visualization graph is presented in Figure 4A. The top 20 most frequently used keywords are shown in Figure 4B. The most commonly occurring keyword, based on our analysis, was “graft versus host disease,” and it was present in 9481 instances. In addition, we stratified keywords into 16 clusters. The top 10 research categories are summarized in Table 4. Hematology (8016, 52.13%) ranked the highest, followed by immunology (5834, 37.94%), transplantation (4737, 30.80%), and oncology (2959, 19.24%).

**Table 4:** The top 10 research categories ranked by count.

Rank	Research Category	TA (%)
1	HEMATOLOGY	8016 (52.13)
2	IMMUNOLOGY	5834 (37.94)
3	TRANSPLANTATION	4737 (30.80)
4	ONCOLOGY	2959 (19.24)
5	BIOPHYSICS	1334 (8.67)
6	MEDICINE RESEARCH EXPERIMENTAL	1087 (7.07)
7	SURGERY	1075 (6.99)
8	CELL BIOLOGY	647 (4.21)
9	PEDIATRICS	573 (3.73)
10	MEDICINE GENERAL INTERNAL	432 (2.81)

### 3.3. Top 10 cited article analysis

The top 10 cited publications are summarized in Table 5. The article with the largest citation number was entitled “Human mesenchymal stem cells modulate allogeneic immune cell responses” and was published in the BLOOD journal in 2005. This study examined the relationship between allogeneic mesenchymal stem cells (MSCs) and immune cells and proposed an underlying mechanism that regulates MSC-driven tolerance, which could be extremely beneficial to the prevention of GVHD, rejection, and inflammation.^[26]^ The next highly cited work was by Ruggeri et al^[27]^ who evaluated the “Effectiveness of donor natural killer cell alloreactivity in mismatched hematopoietic transplants.” The most recent articles, ranking 4th and 6th, were published in 2008.

**Table 5:** The top 10 cited articles on graft versus host disease.

Rank	Article title	Authors	Journal	Publication year	Citations	Citation frequency per year
1	Human mesenchymal stem cells modulate allogeneic immune cell responses	Aggarwal, S; Pittenger, MF	BLOOD	2005	3000	187.5
2	Effectiveness of donor natural killer cell alloreactivity in mismatched hematopoietic transplants	Ruggeri, L; Capanni, M; Urbani, E; Perruccio, K; Shlomchik, WD; Tosti, A; Posati, S; Rogaia, D; Frassoni, F; Aversa, F; Martelli, MF; Velardi, A	SCIENCE	2002	2220	116.84
3	Treatment of severe acute graft-versus-host disease with third party haploidentical mesenchymal stem cells	Le Blanc, K; Rasmusson, I; Sundberg, B; Gotherstrom, C; Hassan, M; Uzunel, M; Ringden, O	LANCET	2004	1943	114.29
4	Mesenchymal stem cells for treatment of steroid-resistant, severe, acute graft-versus-host disease: a phase II study	LeBlanc, K; Frassoni, F; Ball, L; Locatelli, F; Roelofs, H; Lewis, I; Lanino, E; Sundberg, B; Bernardo, ME; Remberger, M; Dini, G; Egeler, RM; Bacigalupo, A; Fibbe, W; Ringden, O	LANCET	2008	1855	142.69
5	Development of lupus-like autoimmune diseases by disruption of the PD-1 gene encoding an ITIM motif-carrying immunoreceptor	Nishimura, H; Nose, M; Hiai, H; Minato, N; Honjo, T	IMMUNITY	1999	1622	73.73
6	Mesenchymal stem cell-mediated immunosuppression occurs via concerted action of chemokines and nitric oxide	Ren, GW; Zhang, LY; Zhao, X; Xu, GW; Zhang, YY; Roberts, AI; Zhao, RC; Shi, YF	CELL STEM CELL	2008	1233	94.85
7	Hematopoietic cell transplantation in older patients with hematologic malignancies: replacing high-dose cytotoxic therapy with graft-versus-tumor effects	McSweeney, PA; Niederwieser, D; Shizuru, JA; Sandmaier, BM; Molina, AJ; Maloney, DG; Chauncey, TR; Gooley, TA; Hegenbart, U; Nash, RA; Radich, J; Wagner, JL; Minor, S; Appelbaum, FR; Bensinger, WI; Bryant, E; Flowers, MED; Georges, GE; Grumet, FC; Kiem, HP; Torok-Storb, B; Yu, G; Blume, KG; Storb, RF	BLOOD	2001	1070	53.5
8	Epidemiology and outcome of mould infections in hematopoietic stem cell transplant recipients	Marr, KA; Carter, RA; Crippa, F; Wald, A; Corey, L	CLINICAL INFECTIOUS DISEASES	2002	1044	54.95
9	CD4(+)CD25(+) regulatory T cells preserve graft-versus-tumor activity while inhibiting graft-versus-host disease after bone marrow transplantation	Edinger, M; Hoffmann, P; Ermann, J; Drago, K; Fathman, CG; Strober, S; Negrin, RS	NATURE MEDICINE	2003	928	51.56
10	Prevention of graft versus host disease by inactivation of host antigen-presenting cells	Shlomchik, WD; Couzens, MS; Tang, CB; McNiff, J; Robert, ME; Liu, JL; Shlomchik, MJ; Emerson, SG	SCIENCE	1999	926	42.09

CD25 = cluster of differentiation 25, CD4 = cluster of differentiation 4, ITIM = immunoreceptor tyrosine-based inhibitory motif, PD-1 = programmed cell death protein 1, TA = total article.

### 3.4. Influential authors’ analysis

We also analyzed which authors contributed the most to the field of GVHD research. The level of influence of each researcher is determined by the number of citations and “ratio of cations.” Among the 64,200 authors in the GVHD research field, 4956 published more than 5 articles that were included in this study. The 10 most productive authors are listed in Table 6. They contributed to 1345 articles (8.75%) on GVHD. Among them, Blazar, BR from the Division of Blood and Marrow Transplantation, Department of Pediatrics, Masonic Cancer Center, University of Minnesota, Minneapolis, United States, contributed the most articles (177 articles), followed by Mohty, M and Socie, G from the Saint-Antoine Hospital, Sorbonne University, France. This study identified some authors, namely Antin, JH, and Martin, PJ, who published relatively fewer articles but received considerably higher citations and, therefore, garnered much popularity and influence.^[22]^ The top 10 most frequently cited articles are summarized in Table 7.

**Table 6:** The top 10 most productive authors.

Rank	Author	N	Total citation	H-index	Country	Affiliation
1	Blazar, BR	177	8515	102	United States	Division of Blood and Marrow Transplantation, Department of Pediatrics, Masonic Cancer Center, University of Minnesota, Minneapolis
2	Mohty, M	167	3753	65	France	Saint-Antoine Hospital, Sorbonne University
3	Socie, G	158	6956	96	France	Saint-Antoine Hospital, Sorbonne University
4	Lee, SJ	133	5771	57	United States	Clinical Research Division, Fred Hutchinson Cancer Research Center; Seattle Cancer Care Alliance; Division of Medical Oncology, Department of Medicine, University of Washington
5	Nagler, A	126	2897	67	Israel	Chaim Sheba Medical Center, Tel Aviv University
6	Huang, XJ	125	2311	31	China	Institute of Hematology, Peking University People’s Hospital
7	Antin, JH	118	6925	98	United States	Department of Hematologic Malignancies, Dana-Farber Cancer Institute, Harvard Medical School
8	Labopin, M	118	2811	41	France	Saint-Antoine Hospital, Sorbonne University
9	Martin, PJ	113	5369	53	United States	Clinical Research Division, Fred Hutchinson Cancer Research Center; Seattle Cancer Care Alliance; Division of Medical Oncology, Department of Medicine, University of Washington
10	Blaise, D	110	2315	53	France	Departement D’Hematologie, Programme de Transplantation et de Therapie Cellulaire, Centre de Recherche en Cancerologie de Marseille, Institut Paoli Calmettes

**Table 7:** The top 10 most cited authors.

Rank	Author	N	Total citation	H-index	Country	Affiliation
1	Blazar, BR	177	8515	102	United States	Division of Blood and Marrow Transplantation, Department of Pediatrics, Masonic Cancer Center, University of Minnesota, Minneapolis
2	Socie, G	158	6956	96	France	Saint-Antoine Hospital, Sorbonne University
3	Antin, JH	118	6925	98	United States	Department of Hematologic Malignancies, Dana-Farber Cancer Institute, Harvard Medical School
4	Weisdorf, DJ	97	6233	81	United States	Division of Blood and Marrow Transplantation, Department of Pediatrics, Masonic Cancer Center, University of Minnesota, Minneapolis
5	Ringden, O	88	5881	76	Sweden	Department of Clinical Science, Intervention and Technology (CLINTEC), Karolinska Institutet
6	Lee, SJ	133	5771	57	United States	Clinical Research Division, Fred Hutchinson Cancer Research Center; Seattle Cancer Care Alliance; Division of Medical Oncology, Department of Medicine, University of Washington
7	Horowitz, MM	63	5628	36	United States	Center for International Blood and Marrow Transplant Research, Milwaukee, Wisconsin; Department of Medicine, Medical College of Wisconsin, Milwaukee, WI
8	Martin, PJ	113	5369	53	United States	Clinical Research Division, Fred Hutchinson Cancer Research Center; Seattle Cancer Care Alliance; Division of Medical Oncology, Department of Medicine, University of Washington
9	Bacigalupo, A	69	5362	69	Italy	Department of Hematology, Fondazione Policlinico Universitario Gemelli Istituto di ricovero e cura a carattere scientifico, Universita’ Cattolica del Sacro Cuore
10	Soiffer, RJ	98	5133	95	United States	Clinical Research Division, Fred Hutchinson Cancer Research Center; Seattle Cancer Care Alliance; Division of Medical Oncology, Department of Medicine, University of Washington

## 4. Discussion

### 4.1. Overview

In this study, we analyzed the published literature on GVHD. Recent decades have seen an explosion in GVHD research, covering a wide range of academic journals,^[28]^ thus reflecting the significance of GVHD research. The conclusions of this study will be highly beneficial to scientists involved in GVHD research and will further progress and collaborate within this field.

With advancements in GVHD research, a growing number of studies have been published each year. The first GVHD article was published in 1977 and was entitled “Reduction of Fetal Graft Versus Host Disease by H-3 Thymidine Suicide of Donor Cells Cultured with Host Cells” and published in the *Transplantation* journal. In this article, the authors proposed the use of the 3H-TdR suicide technique to dramatically reduce the induction of GVHD.^[29]^ However, until 1994, no more articles and/or related research had been published on GVHD based on the WoS database. The massive increase in GVHD publications began in 1999 and has maintained steady growth. Significant advancements in this field may, in part, be related to the success and progression of HSCT. With improved technology, an increasing number of patients choose the HSCT technology, and the complications related to this procedure are starting to surface. Unfortunately, regardless of advances in donor selection, conditioning regimens, and greater availability of allograft sources, transplant recipients still experience morbidity and mortality related to GVHD.^[30]^ Moreover, GVHD can originate not only from malignant hemopathy but also from other diseases that require organ transplantation. Therefore, to deepen our understanding of GVHD, scientists have conducted research using numerous independent design strategies to improve surgical outcomes and general quality of life.

Our analyses of the most prolific countries and institutions confirmed that GVHD research is being conducted worldwide. More importantly, first-world countries such as the United States contribute the most to GVHD research. This may be due to the fact that the first article on GVHD came out of the United States, and since then, the National Institutes of Health has been very supportive of research in this field.^[31]^ Our analysis also highlights the lack of considerable contribution from developing countries. The journals listed in Table 1, such as *Biology of Blood and Marrow Transplantation*, *Bone Marrow Transplantation*, and *British Journal of Hematology*, are core journals that published articles on GVHD research. Further studies can serve as guidance for submitting future work to those journals.^[32]^

The top 10 most cited articles had JIF quartiles of Q1. These studies were published more than 5 years ago, suggesting that they have provided significant research information. For comparison, the most cited article was cited 3000 times. Highly cited articles mostly discuss the underlying mechanism(s) of GVHD. For instance, the 2nd most cited article proposed that the infusion of alloreactive natural killer cells prior to transplantation eliminates the high-intensity conditioning requirement and simultaneously diminishes the incidence of murine GVHD.^[27]^ This category of research was comparable with the articles that held 5th and 9th place, in the most cited list. The 5th highly cited article established a programmed cell death protein 1 null mutation in 2C T cell receptor transgenic mice of the H-2b/d background and revealed that the mice developed chronic and systemic GVHD.^[33]^ The 9th most cited article showed that CD4^+^CD25^+^ T cells are strong regulators of GVHD versus conventional donor T cell-mediated graft versus tumor activity.^[34]^ Finally, some articles were based on clinical treatments and clinical reports,^[35,36]^ namely, the 3rd and 4th highly cited articles, which focused on the clinical aspects of GVHD therapy.

Our analyses and conclusions hold great significance because they offer an exhaustive overview of the current status and direction of GVHD research. Moreover, this comprehensive study will aid scientists in forming beneficial collaborations that will significantly enhance research in this field.

### 4.2. Limitation and suggestion for future research

Our study has certain limitations. First, research on GVHD was restricted to publications available in the WoS. The use of only 1 database limited the comprehensiveness of the study, despite the quality of the article sources. Second, we extracted only articles that included the terms of the research strategy. This may have introduced unintentional selection bias. Third, this study was restricted to articles published in English. Ideally, a comprehensive search should include as many possible source types. Finally, certain factors, such as the duration of studies and works published after April 2021, may skew the true research conditions and bibliometric results. In future studies, individual clusters should be examined in detail to gain a comprehensive understanding of GVHD research.

## 5. Conclusion

In this study, we compared articles published from 1977 to 2021 to provide comprehensive knowledge of GVHD research for interested scientists. Based on our findings, developing nations are lagging in GVHD research and need to increase their efforts in this field. Developed countries play a significant role in terms of publications, organizations/institutions, and funding agencies. Our work can provide an avenue for scientists to collaborate with influential authors and organizations.

### Author contributions

All the authors made substantial contributions to conception and design, acquisition of data, or analysis and interpretation of data; involved in drafting the article or revising it critically for important intellectual content; gave final approval of the version to be published. Each author should have participated sufficiently in the work to take public responsibility for appropriate portions of the content; agreed to be accountable for all aspects of the work in ensuring that questions related to the accuracy or integrity of any part of the work are appropriately investigated and resolved.
